# Peroxisome-driven ether-linked phospholipids biosynthesis is essential for ferroptosis

**DOI:** 10.1038/s41418-021-00769-0

**Published:** 2021-03-17

**Authors:** Weiwei Cui, Dong Liu, Wei Gu, Bo Chu

**Affiliations:** 1 Department of Cell Biology, School of Basic Medical Sciences, Cheeloo College of Medicine, Shandong University, Jinan, Shandong China; 2 Institute for Cancer Genetics, and Department of Pathology and Cell Biology, and Herbert Irving Comprehensive Cancer Center, College of Physicians & Surgeons, Columbia University, New York, NY USA

**Keywords:** Phospholipids, Cancer metabolism

## Abstract

It is well established that ferroptosis is primarily induced by peroxidation of long-chain poly-unsaturated fatty acid (PUFA) through nonenzymatic oxidation by free radicals or enzymatic stimulation of lipoxygenase. Although there is emerging evidence that long-chain saturated fatty acid (SFA) might be implicated in ferroptosis, it remains unclear whether and how SFA participates in the process of ferroptosis. Using endogenous metabolites and genome-wide CRISPR screening, we have identified FAR1 as a critical factor for SFA-mediated ferroptosis. FAR1 catalyzes the reduction of C16 or C18 saturated fatty acid to fatty alcohol, which is required for the synthesis of alkyl-ether lipids and plasmalogens. Inactivation of FAR1 diminishes SFA-dependent ferroptosis. Furthermore, FAR1-mediated ferroptosis is dependent on peroxisome-driven ether phospholipid biosynthesis. Strikingly, TMEM189, a newly identified gene which introduces vinyl-ether double bond into alkyl-ether lipids to generate plasmalogens abrogates FAR1-alkyl-ether lipids axis induced ferroptosis. Our study reveals a new FAR1-ether lipids-TMEM189 axis dependent ferroptosis pathway and suggests TMEM189 as a promising druggable target for anticancer therapy.

Ether phospholipids represent an important group of phospholipids containing a glycerol backbone with an alkyl or a vinyl bond connecting a fatty alcohol at sn-1 position, usually polyunsaturated fatty acid (PUFA) including docosahexaenoic acid and arachidonic acid at sn-2. Ether phospholipids are initially synthesized in peroxisomes and processed in the endoplasmic reticulum (ER) [1–3]. Plasmalogens are the most abundant form of ether phospholipids which have a vinyl ether bond, enriched in the brain and heart tissues [1–3]. The plasmalogens have been found as endogenous antioxidants with vinyl ether bond susceptible to cleavage by reactive oxygen species (ROS). The deficiency of plasmalogens correlates with various human disorders, including Alzheimer’s disease and cancer [1, 2, 4].

Ferroptosis is an iron-dependent form of non-apoptotic cell death induced by excess accumulation of peroxidized phopholipids, generated through oxidation of the PUFA moieties at sn-2 position of membrane phospholipids [5–9]. Ferroptosis is morphologically, biochemically and genetically distinct from other forms of cells death [5], which is tightly regulated by glutathione peroxidase 4 (GPX4) via converting lipid hydroperoxides (PUFA-OOH) into non-toxic lipid alcohols (PUFA-OH) [10, 11]. Emerging evidence indicates that ferroptosis is implicated in ischemia–reperfusion injury (IRI), neurodegeneration, antiviral immunity, cancer immunotherapy and tumor suppression [11–19].

Accumulating evidence reveals a robust link between lipid metabolism and ferroptosis [14, 20–24]. However, little is known about the role of ether phospholipids in ferroptosis. In the present study, we revealed the FAR1-TMEM189 axis as a central pathway to drive the susceptibility of ferroptosis. FAR1-TMEM189 axis specifically synthesizes alkyl and vinyl ether phospholipid, where the two isoforms of ether phospholipid play distinct role in ferroptosis. Our findings provide an insight into the mechanism of ether phospholipid-mediated ferroptosis, with implications for novel treatment options for cancer therapy.

## Results

### Identification and validation of 1-hexadecanol as a ferroptosis activator

**Fig. 1:**
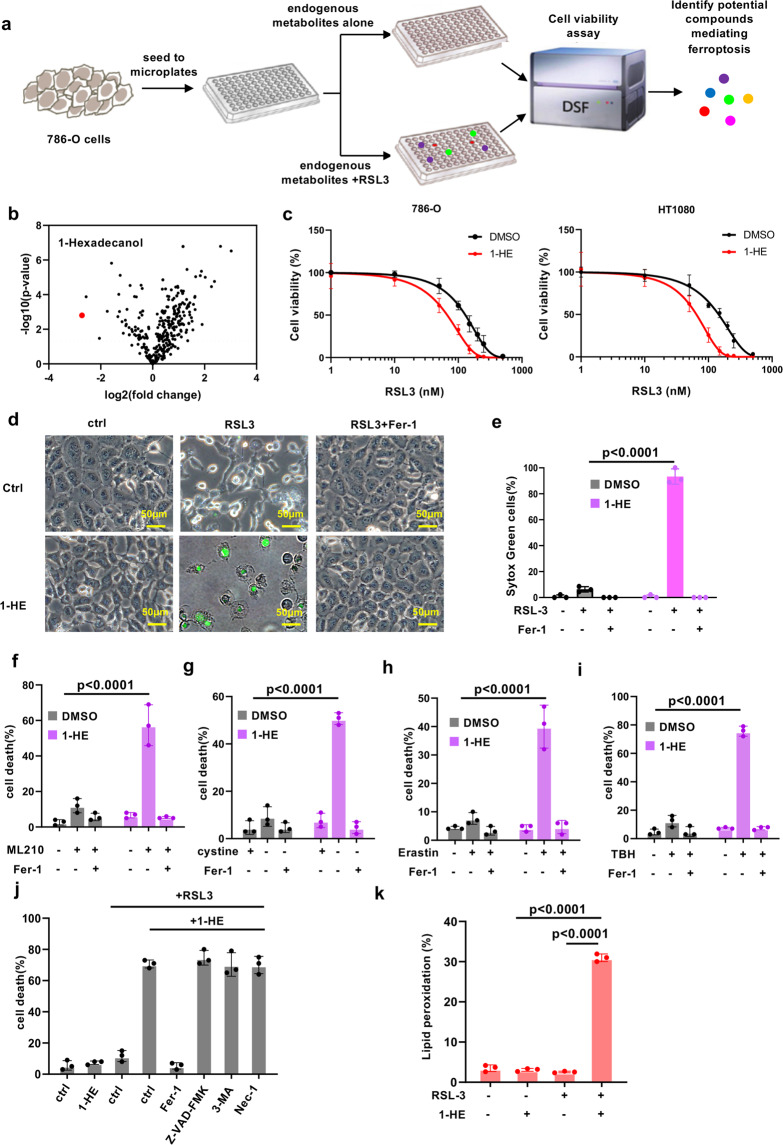
Identification and validation of 1-hexadecanol as a ferroptosis inducer. **a** Schematic of the identification of potential ferroptosis-induced endogenous metabolites, using 786-O cells pretreated with endogenous metabolites followed by RSL3 treatment for 24 h. **b** Volcano plots showing the top hits in endogenous metabolites treated screen in 786-O cells, highlighting 1-hexadecanol as a ferroptosis activator. **c** Dose-dependent toxicity of RSL-3 induced cell death of 786-O and HT1080 cells treated with 1-hexadecanol (20μM). Cell viability was assessed 24 h thereafter using CCK8. **d** Representative phase-contrast images of HT1080 cells pre-incubated with 1-hexadecanol (20 μM) for 12 h were treated with RSL3 (200 nM) and Fer-1 (1 μM). Dead cells were stained with Sytox Green. Scale bars, 100 µm.The experiments were repeated twice, independently, with similar results. **e** Cell death measurement of HT1080 cells pre-incubated with 1-hexadecanol (20 μM) for 12 h were treated with RSL3 (200 nM) and Fer-1 (1 μM) for 12 h. Cell death measurement of HT1080 cells pre-incubated with 1-hexadecanol (20 μM) for 12 h were treated with 200 nM of ML210 (**f**), cystine-free medium (**g**), 10 μM of erastin (**h**) or 100uM of TBH (**i**) for 12 h. **j** Cell death measurement of HT1080 cells pre-incubated with 1-hexadecanol (20 μM) for 12 h were treated with RSL3 (200 nM) and indicated inhibitors. Fer-1, 1 μM ferrostatin-1; NAC, 5 mM; Nec, 2 μM necrostatin-1; 20 μM Z-VAD-FMK. **k** Lipid peroxidation measurements in HT1080 cells pre-incubated with 1-hexadecanol (20 μM) for 12 h were treated with RSL3 (200 nM) for 4 h. Data and error bars are mean ± s.d., *n* = 3 (**e**–**k**) independent repeats. All *P* values were calculated using two-tailed unpaired Student’s *t*-test.

Although recent studies have been reported the endogenous metabolites in the glucose metabolism pathway are implicated in ferroptosis besides cysteine-GSH pathway [24, 25], it is little known whether a network system of metabolites regulates ferroptosis. There is a tight correlation between glucose, lipid and amino acid metabolism, suggesting that there are alternative metabolites involved in the process of ferroptosis. To uncover these metabolites, we performed a screening library of 350 small molecules of metabolites for ferroptosis assay in 786-O cells (Fig. 1a). Screening of this library identified 1-hexadecanol (1-HE), which is the most potent metabolite promoting GPX4 inhibitor RSL-3 induced ferroptosis (Fig. 1b). To validate this finding, 786-O and HT1080 cells were utilized to test the effect of 1-HE. The cells treated with 1-HE were particularly sensitive to ferroptosis (Fig. 1c, Supplementary Fig. S1a). 1-HE mediated cell death was completely blocked by ferroptosis inhibitor ferrostatin-1 (Fer-1) but not by the apoptosis inhibitor carbobenzoxy-valyl-alanyl-aspartyl-[O-methyl]-fluoromethylketone (Z-VAD-FMK), the necroptosis inhibitor necrostatin-1 or autophagy inhibitor 3-MA (Fig. 1d, e, j, Supplementary Fig. S1b). 1-HE mediated ferroptosis can also be induced by another GPX4 inhibitor ML210, cystine starvation, erastin-suppressed cysteine uptake or TBH-induced ROS (Fig. 1f–i). As lipid peroxidation is a hallmark of ferroptosis, we next estimated the levels of lipid peroxidation in 1-HE pre-incubated cells upon RSL3 treatment using BODIPY-C11 staining. As expected, high level of lipid peroxidation was detected in 1-HE treated cells (Fig. 1k). Taken together, these data indicate that 1-HE is a metabolite strongly enhancing the vulnerability to ferroptosis.

### FAR1 is critical for saturated fatty acid-induced ferroptosis

1-HE is saturated fatty alcohol derived from the reduction of palmitic acid (PA). As 1-HE promotes ferroptotic cell death, we have a curiosity about whether C16 or C18 saturated fatty acid (SFA) or fatty alcohol sensitizes the cells to ferroptosis. Interestingly, high levels of cell death were observed in C18:0 fatty alcohol (1- Octadecanol,1-OE) and SFAs including C16:0 PA and C18:0 stearic acid (SA) treatment while there was no obvious cell death in DMSO-treated cells (Supplementary Fig. S1c–f). Notably, fatty alcohol exhibited higher ability to sensitize the cells to ferroptosis than SFA (Supplementary Fig. S1c–f). These aforementioned data prompted us to further examine how SFA increased the susceptibility of ferroptosis and why fatty alcohol displays stronger effect on ferroptosis induction.

**Fig. 2:**
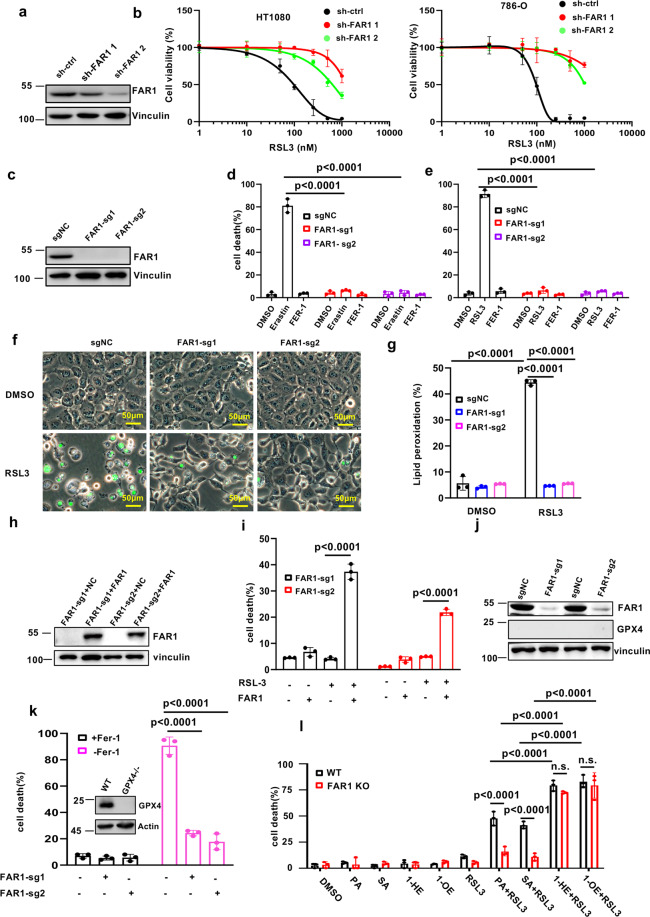
FAR1 is critical for long-chain saturated fatty acid-induced ferroptosis. **a** Western blot analysis of HT1080 cells expressing shRNA-ctrl or shRNA-FAR1. The experiments were repeated twice, independently, with similar results. **b** Dose-dependent toxicity of RSL-3 induced cell death of HT1080 and 786-O cells expressing shRNA-ctrl or shRNA-FAR1. Cell viability was assessed 24 h thereafter using CCK8. **c** Western blot analysis of HT1080 cells expressing sg-ctrl or sg-FAR1. The experiments were repeated twice, independently, with similar results. **d** Cell death measurement of HT1080 cells expressing sg-ctrl or sg-FAR1 treated with erastin (10uM) and Fer-1 (1 μM) for 24 h. **e** Cell death measurement of HT1080 cells expressing sg-ctrl or sg-FAR1 treated with RSL3 (500 nM) and Fer-1 (1 μM) for 12 h. **f** Representative phase-contrast images of HT1080 cells expressing sg-ctrl or sg-FAR1 treated with RSL3 (500 nM). Dead cells were stained with Sytox Green. Scale bars, 100 µm.The experiments were repeated twice, independently, with similar results. **g** Lipid peroxidation measurements in HT1080 cells expressing sg-ctrl or sg-FAR1 treated with erastin (10 μM). **h** Western blot analysis of HT1080 cells expressing sg-ctrl or sg-FAR1 transfected with FAR1 plasmids. The experiments were repeated twice, independently, with similar results. **i** Cell death measurements of HT1080 cells expressing sg-FAR1 transfected with FAR1. **j** Western blot analysis of HT1080 GPX4−/− cells expressing sg-ctrl or sg-FAR1. The experiments were repeated twice, independently, with similar results. **k** Cell death measurements of HT1080 GPX4−/− cells expressing sg-ctrl or sg-FAR1 upon Fer-1 withdraw. **l** Cell death measurements of WT and FAR1 KO HT1080 cells were pre-incubated with PA (10 μM), SA (10 μM), 1-HE (10 μM) and 1-OE (10uM) for 12 h, and then treated with RSL3(100 nM). PA, palmitic acid. 1-HE, 1-hexadenocal. SA, stearic acid. 1-OE, 1-Octadecanol. Data and error bars are mean ± s.d., *n* = 3 (**b**, **d**, **e**, **g**, **i**, **k**–**l**) independent repeats. All *P* values were calculated using two-tailed unpaired Student’s *t*-test.

Previous study showed that the peroxisomal enzyme fatty acyl-CoA reductase FAR1 is required for the formation of fatty alcohol via reduction of SFA [26, 27]. To validate whether FAR1-mediated fatty alcohol synthesis is required for ferroptosis, we silenced the gene expression with independent FAR1 shRNAs. As shown in Fig. 2a, FAR1 protein levels were largely abrogated in HT1080 cells. As expected, ferroptosis was readily induced in the cells expressing sh-ctrl but not sh-FAR1 (Fig. 2b). To further confirm this phenotype, we generated FAR1−/− HT1080 cells using two independent sgRNAs (Fig. 2c). Indeed, given their lack of FAR1 expression, these cells are robustly resistant to erastin or RSL3-induced ferroptosis (Fig. 2d–f). BODIPY-C11 staining reveals that elevated levels of endogenous lipid peroxidation were detected in WT but not FAR1−/− cells (Fig. 2g). Moreover, RSL3-induced ferroptosis in FAR1-null HT1080 cells was restored by ectopic expression of wildtype FAR1 (Fig. 2h, i). Notably, eliminating FAR1 level in GPX4−/− cells by FAR1 sgRNA rendered the cells resistant to ferroptosis withdrawn Fer-1 (Fig. 2j, k). In addition, 1-HE and 1-OE significantly sensitized FAR1−/− cells to ferroptosis, while no obvious cell death was observed in PA and SA pre-incubated FAR1−/− cells (Fig. 2l). Taken together, these data reveal that SFA-mediated ferroptosis is dependent of FAR1 function essential for fatty alcohol generation.

### FAR1-mediated ferroptosis is dependent on peroxisome-driven ether phospholipid synthesis

**Fig. 3:**
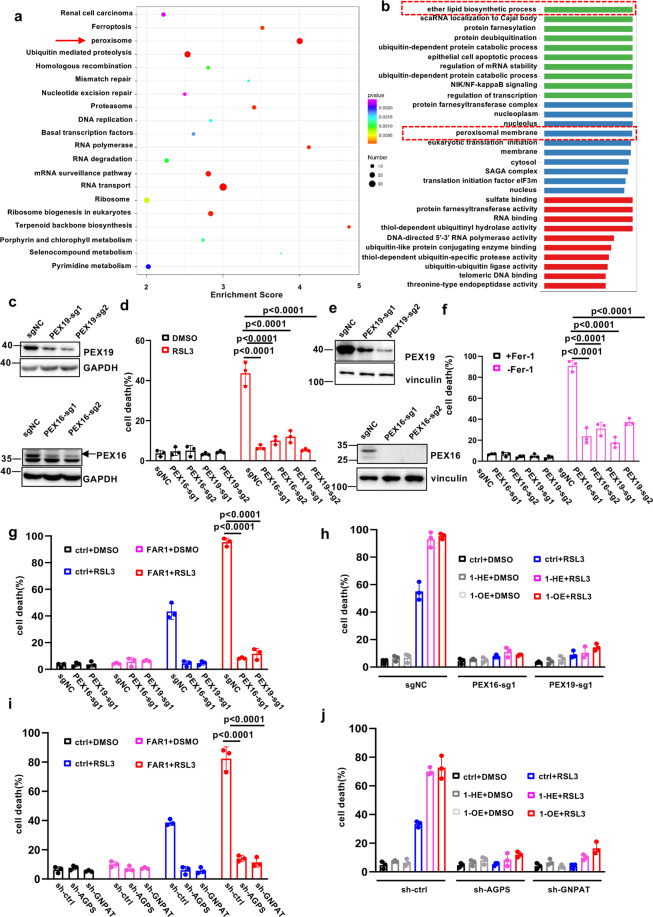
FAR1-mediated ferroptosis is dependent on peroxisome-driven ether phospholipid synthesis. **a** Kyoto Encyclopedia of Genes and Genomes (KEGG) pathway enrichment analysis of the identified differentially expressed genes between DMSO and ML210 treated 786-O cells which are transfected with a genome-wide CRISPR-CAS9 sgRNA lentivirus pools. The twenty of most significantly enriched pathways (*P* < 0.05 by Fisher’s exact test) are shown, highlighting peroxisome pathway. **b** The Gene Ontology (GO) analysis showing that peroxisome membrane proteins and ether phospholipid biosynthetic process are involved in the process of ferroptosis. **c** Western blot analysis of HT1080 cells expressing sg-ctrl, sg-PEX16 or sg-PEX19. The experiments were repeated twice, independently, with similar results. **d** Cell death measurement of HT1080 cells expressing sg-ctrl sg-PEX16 or sg-PEX19 treated with RSL3 (500 nM) for 12 h. **e** Western blot analysis of HT1080 GPX4−/− cells expressing sg-ctrl, sg-PEX16 or sg-PEX19. The experiments were repeated twice, independently, with similar results. **f** Cell death measurements of HT1080 GPX4−/− cells expressing sg-ctrl, sg-PEX16, or sg-PEX19 upon Fer-1 withdrawal. **g** Cell death measurement of HT1080 cells expressing sg-ctrl sg-PEX16 or sg-PEX19 transfected with FAR1 treated with RSL3 (200 nM) for 12 h. **h** Cell death measurement of HT1080 cells expressing sg-ctrl sg-PEX16 or sg-PEX19 pre-incubated with 1-HE (20 μM) or 1-OE (20 μM) treated with RSL3 (500 nM) for 12 h. **i** Cell death measurement of HT1080 cells expressing sh-ctrl sh-GNPAT or sh-AGPS transfected with FAR1 treated with RSL3 (500 nM) for 12 h. **j** Cell death measurement of HT1080 cells expressing sh-ctrl sh-GNPAT or sh-AGPS pre-incubated with 1-HE (20 μM) or 1-Oct (20 μM) treated with RSL3 (200 nM) for 12 h. Data and error bars are mean ± s.d., *n* = 3 (**d**, **f**–**i**) independent repeats. All *P* values were calculated using two-tailed unpaired Student’s *t*-test.

As FAR1 is a key rate-limiting enzyme to synthesize ether phospholipid by supplying for fatty alcohol [26, 27], we tested whether ether phospholipid biosynthesis pathway is involved in ferroptosis. Using a public genome-wide CRISPR-CAS9 screening in DMSO and ML210 treated 786-O cells [23], we performed KEGG analysis of the most differentially expressed genes during occurrence of ferroptosis and highlighted peroxisome as the most enriched pathway on the basis of the number of changed genes and statistical significance (Fig. 3a, Supplementary Fig. S2a). Notably, the peroxisome pathway also stood out in the GO analysis, as well for ether phospholipid biosynthesis pathway (Fig. 3b, Supplementary Fig. S2b). In addition, we found that the ether phospholipid synthesis-related genes FAR1, GNPAT and AGPS (Supplementary Fig. S2c), accompanied with peroxisome genes PEX3, PEX7, PEX16, and PEX19 were highlighted as the most enriched CRISPR hits (Supplementary Fig. S2d).

As ether lipids which contain two subtypes of alky and vinyl-ether (Supplementary Fig. S2f) are initially synthesized in peroxisome, we firstly tested whether the peroxisomes have any effects on ferroptosis. PEX16 and PEX19 are critical for peroxisome de novo biogenesis (Supplementary Fig. S2e), loss of any of these genes results in the cells deficient in peroxisome structures [28, 29]. Therefore, we eliminated the peroxisomes in HT1080 cells expressing PEX16 or PEX19 sgRNAs. As shown in Fig. 3c, PEX16 and 19 protein levels were significantly reduced by expressing sgRNA. In the meanwhile, PEX16/19 knockout induced a significant reduction of the abundance of peroxisomes (Supplementary Fig. S3a). Notably, the cells expressing PEX sgRNAs exhibited a robust resistance to RSL-3 induced ferroptosis (Fig. 3d). In addition, little cell death was observed in HT1080 GPX4−/− cells transfected with PEX sgRNAs upon Fer-1 withdrawal (Fig. 3e, f). To further validate this finding, we examined whether FAR1 promotes ferroptosis in the cells lack of peroxisomes. Interestingly, although FAR1 was over-expressed in the cells expressing PEX sgRNAs, no obvious cell death was observed in these cells upon RSL3 treatment (Fig. 3g). In addition, 1-HE and 1-OE lose the effect to promote the sensitivity to ferroptosis when peroxisomes are lost in the cells (Fig. 3h). In accordance with these findings, knockdown of GNPAT and AGPS significantly suppressed ferroptotic cell death (Supplementary Fig. S2g–j). Notably, the resistance to ferroptosis was not rescued by ectopic expression of FAR1 (Fig. 3i). Moreover, the addition of fatty alcohol into cells expressing GNPAT or AGPS shRNA has no effect on RSL3-induced ferroptosis (Fig. 3j). To further demonstrate the role of ether phospholipids, WT and FAR1−/− HT1080 cells were supplemented with alkyl-ether lipids C16(-O-)-20:4 PC, we observed similarly high levels of cell death in these two cell lines (Supplementary Fig. S3b). These data demonstrate that ether phospholipid synthesis is crucial for ferroptosis and this effect is dependent of the functions of peroxisomes.

### FAR1 is required for ferroptosis response in a panel of cancer cell lines

**Fig. 4:**
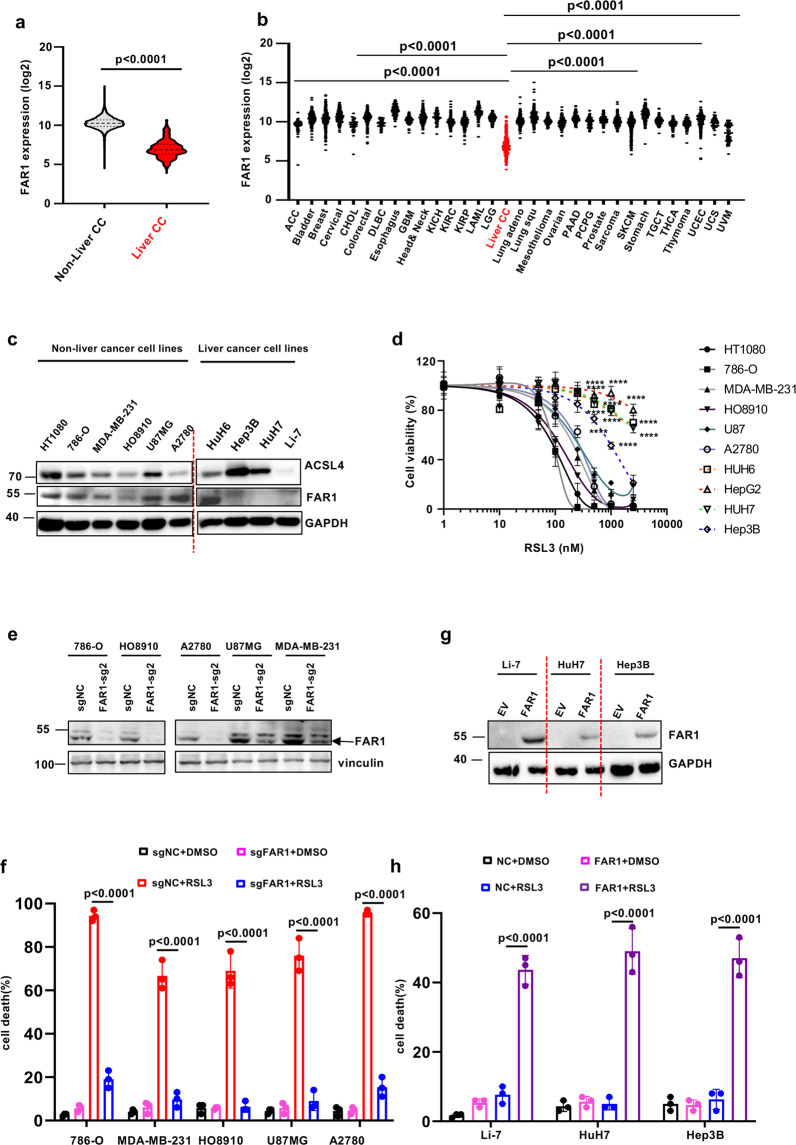
FAR1 expression levels are positively correlated with ferroptosis response in a panel of cancer cell lines. **a** FAR1 mRNA expression levels in liver carcinoma (liver-CC) and non-liver carcinoma tumor samples from TCGA RNA-Seq database. *P* values were calculated using two-tailed unpaired Student’s *t*-test. **b** FAR1 mRNA expression levels in a series of cancer types from TCGA RNA-Seq database. **c** Western blot analysis of a panel of cancer cell lines. The Western blot experiments were repeated twice, independently, with similar results. **d** Dose-dependent toxicity of RSL-3 induced cell death in a panel of cancer cell lines. Cell viability was assessed 24 h thereafter using CCK8. **e** Western blot analysis of cancer cell lines expressing sg-ctrl or sg-FAR1. The Western blot experiments were repeated twice, independently, with similar results. **f** Cell death of cancer cell lines expressing sg-ctrl or sg-FAR1 treated with RSL3. **g** Western blot analysis of liver cancer cell lines over-expressing FAR1. The Western blot experiments were repeated twice, independently, with similar results. **h** Cell death of liver cancer cell lines over-expressing FAR1 treated with RSL3. Data and error bars are mean ± s.d., *n* = 3 (**d**, **h**) independent repeats. All *P* values were calculated using two-tailed unpaired Student’s *t*-test.

Since FAR1 is essential for ether lipid generation which sensitizes the cells to ferroptosis, we then sought to determine whether FAR1 functions as a tumor suppressor by activating ferroptotic responses. Strikingly, TCGA gene enrichment analysis reveals that FAR1 exhibits a significantly lower enrichment in liver cancer than in other types of cancer (Fig. 4a, b). In line with this finding, Cancer Cell Line Encyclopedia (CCLE) database analysis also demonstrates FAR1 expression is specifically lower in liver cancer cell lines (Supplementary Fig. S4a). To further validate these findings, we examined FAR1 protein levels in a variety of human cancer cell lines. As shown in Fig. 4c, FAR1 protein levels were barely undetectable in liver cancer cell lines while other cancer cell lines exhibited higher FAR1 levels. Interestingly, ferroptosis was readily induced in the cells expressing high levels of FAR1 (Fig. 4d). In contrast, almost all experimental liver cancer cell lines were not susceptible to RSL3-induced ferroptosis (Fig. 4d). These data imply that FAR1 is tightly correlated with ferroptosis in a panel of cancer cell lines.

To further verify the aforementioned findings, we generated ferroptosis-sensitive cancer cell lines expressing FAR1 sgRNAs. As expected, FAR1 protein levels are largely diminished in these cell lines (Fig. 4e). Notably, RSL3-induced ferroptosis was almost completely abolished in these cancer cell lines with low abundant FAR1 (Fig. 4f). Moreover, ectopic expression of FAR1 rendered the liver cancer cell lines susceptible to ferroptosis (Fig. 4g, h). Additionally, analysis of the cancer dependency map (DepMap; https://depmap.org/portal/) revealed that the expression levels of FAR1 are positively correlated with AGPS and acyl-CoA synthetase long-chain family member 4 (ACSL4) (Supplementary Fig. S4b), which incorporates free lipid acids such as PUFAs into phospholipids (PL-PUFAs) [20, 21]. Taken together, these findings demonstrate the specific requirement of FAR1 for ferroptotic cell death in cancer cells.

### FAR1 expression is positively correlated with the process of ferroptosis in renal IRI and tumors

**Fig. 5:**
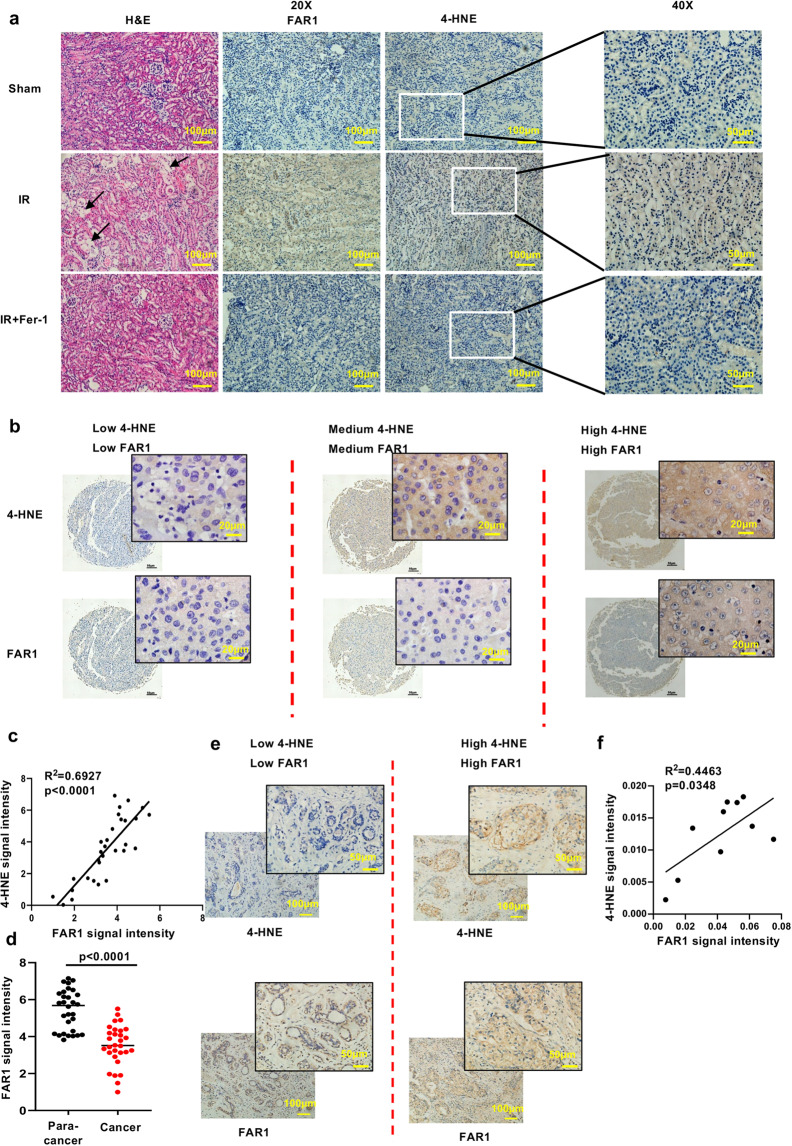
FAR1 responds to the process of ferroptosis in renal IR and tumors. **a** Representative immunohistochemical staining (IHC) of cortical renal tissues from the mice after ischemia reperfusion (IR) or sham treatment. The experiment was repeated three times, independently, with similar results. **b** Representative immunohistochemical staining (IHC) of liver cancer samples. The experiment was repeated twice, independently, with similar results. **c** Dot plot depicting the relevance of the signal intensity of FAR1 and 4-HNE IHC staining in the indicated liver cancer samples. **d** FAR1 signaling intensity in para-cancer and liver cancer tiusses indicates FAR1 is lower expressed in liver cancer tissues. **e** Representative immunohistochemical staining (IHC) of FAR1 and 4-HNE in human breast cancer samples. The experiment was repeated twice, independently, with similar results. **f** Dot plot depicting the relevance of the signal intensity of FAR1 and 4-HNE IHC staining in the breast cancer samples.

Our findings from cancer cell line studies prompted further analysis of FAR1 function in ferroptosis-associated pathological conditions in vivo. As previous studies indicate an important role of ferroptosis in renal IRI [16, 18, 24], we treated WT c57BL/6 mice with vehicle and ferrostatin-1 (as a positive control), and then subjected the mice to an established renal IRI protocol to examine whether FAR1 responds to the process of renal IRI. As expected, immunohistochemical analysis of 4-hydroxynonenalince (4-HNE) staining, which is a molecular marker of ferroptosis [24, 30] reveals strong staining of lipid peroxidation induced by renal IRI (Fig. 5a). Notably, FAR1 exhibited a similar response to renal-IRI-induced ferroptosis (Fig. 5a, Supplementary Fig. S5a). This observation was further confirmed with immunofluorescence analysis using transferrin receptor 1 (TFR1) antibody, a specific ferroptosis marker [30] (Supplementary Fig. S5a). In addition, renal IRI-induced 4-HNE staining and FAR1 expression were largely abolished by ferrostatin-1. Together, these in vivo data suggest that FAR1 is an important gene responsive to hypoxia or oxidative stress-induced ferroptosis.

We next examined whether these findings can be also observed in human cancers. Since the above data (Fig. 4a, b, c) indicated FAR1 is lower expressed in liver cancers, we employed liver cancer as the model to test whether FAR1 is significantly up-regulated during ferroptosis. As expected, IHC staining of FAR1 was highly consistent with 4-HNE staining in the human liver cancer tissue microarray (Fig. 5b, c). Notably, FAR1 signaling intensity is lower in liver cancer than para-cancer tissues (Fig. 5d), indicating FAR1 is a potential tumor suppressor. Moreover, through the gene expression analysis of The Cancer Genome Atlas (TCGA; https://portal.gdc.cancer.gov/) database, we found that FAR1 expression positively correlates with ferroptosis response signature which is accompanied with high ACSL4 and low GPX4 expression levels in colorectal and breast cancers (Supplementary Fig. S4c, d). Further analysis reveals the colorectal and breast cancer patients expressing higher levels of FAR1 have a significantly higher overall survival compared to patients bearing tumors expressing lower levels of FAR1 expression (Supplementary Fig. S4e). IHC staining revealed similar strong relevance of FAR1 and 4-HNE in breast cancers (Fig. 5e, f), indicating FAR1 participates in the process of ferroptosis in human cancers. In addition, comparing to more malignant tumor progression of T3 and T4 stage, the expression levels of FAR1 were much lower in T1 and T2 stage according to tumor nude metastasis (TNM) subsets in breast cancers (Supplementary Fig. S4f). Moreover, Kaplan–Meier survival analysis demonstrated that high expression of FAR1 is associated with improved overall survival in patients with other types of cancers including liver cancer, rectum adenocarcinoma and bladder carcinoma (Supplementary Fig. S4g). Taken together, these data demonstrate that FAR1 is positively correlated with ferroptosis responses in renal IRI and tumors, implying a potential role of FAR1 as a tumor suppresser via ferroptosis.

### TMEM189 promotes ferroptosis resistance via inhibiting expression of FAR1 through plasmalogen synthesis

Previous studies indicate that plasmalogens degrades FAR1 [31], dysregulation of plasmalogens is closely associated with cancer and neurodegeneration including Alzheimer’s disease. This implies inhibition of plasmalogens synthesis might render the cells to ferroptosis. To validate this hypothesis, we focused on plasmanylehtanolamine desaturase TMEM189 gene which is a newly identified gene specifically required for generation of plasmalogens from alkyl ether lipids [32, 33].

**Fig. 6:**
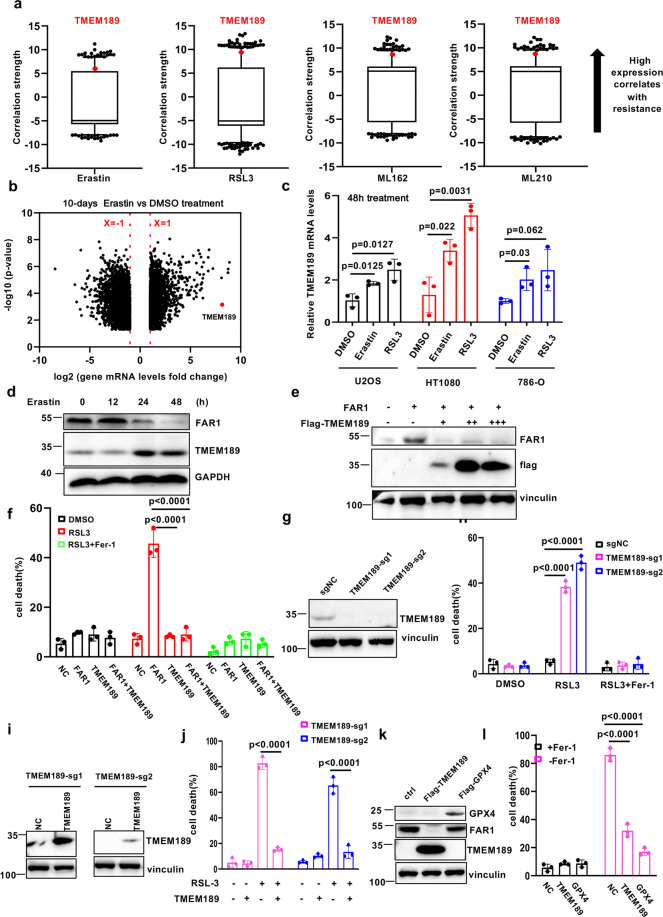
TMEM189 promotes ferroptosis resistance via degradation of FAR1 through plasmalogen synthesis. **a** Box-and-whisker plots show 1st and 99th percentile outlier transcripts (black and red dots) whose expression levels are correlated with cell line sensitivity to indicated ferroptosis inducer. Plotted values are z-scored Pearson’s correlation coefficients. Line, median; box, 10th–90th percentiles. **b** Volcano plots showing the identified differentially expressed genes between DMSO and erastin treated U2OS cells RNA-Seq database. **c** qRT-PCR analysis of TMEM189 mRNA levels in U2OS, HT1080 and 786-O cells treated with erastin and RSL3 for the indicated time. **d** Western blot analysis of HT1080 cells treated with erastin for the indicated time. **e** Western blot analysis of HT1080 cells transfected with FAR1 and TMEM189. **f** Cell death measurement of HT1080 cells transfected with FAR1 and TMEM189 treated with RSL3 (200 nM) for 12 h. **g** Western blot analysis of HT1080 cells expressing sg-ctrl or sg-TMEM189. **h** Cell death measurement of HT1080 cells expressing sg-ctrl or sg-TMEM189 treated with RSL3 (200 nM) for 12 h. **i** Western blot analysis of HT1080 cells expressing sg-ctrl or sg-TMEM189 transfected with TMEM189 plasmids. **j** Cell death measurement of HT1080 cells expressing sg-ctrl or sg-TMEM189 transfected with TMEM189 treated with RSL3 (200 nM) for 12 h. **k** Western blot analysis of HT1080 GPX4−/− cells with ectopic expression of TMEM189. The experiments were repeated twice, independently, with similar results. **l**, Cell death measurement of HT1080 GPX4−/− cells with ectopic expression of TMEM189 upon Fer-1 withdrawal. Data and Error bars are mean ± s.d., *n* = 3 **(c**, **f**, **h**–**j**, **l**) independent repeats. All *P* values were calculated using two-tailed unpaired Student’s *t*-test. All western blot experiments in **d**, **e**, **g**, **i**, **k** were repeated twice, independently, with similar results.

By analyzing data from the Cancer Therapeutics Response Portal (CTRP; http://portals.broadinstitute.org/ctrp/) and evaluating correlations between TMEM189 gene expression profiles across 654 cancer cell lines and cell sensitivities to ferroptosis-inducing agents, we found that TMEM189 was strongly correlated with resistance to ferroptosis inducers (Fig. 6a). Notably, TMEM189 was highlighted as the most differentially upregulated expressed gene in the 10 days DMSO and erastin-treated U2OS cells (Fig. 6b). This observation was confirmed by real-time PCR (Fig. 6c, Supplementary Fig. S5b, c) in U2OS, 786-O and HT1080 cells upon erastin and RSL3 treatment and western blot in HT1080 cells (Fig. 6d). Surprisingly, we observed a dramatic decrease of FAR1 protein levels, accompanied with elevated TMEM189 expression upon erastin treatment (Fig. 6d). In addition, ectopic expression of TMEM189 significantly eliminated FAR1 protein levels and completely abolished FAR1-mediated ferroptosis (Fig. 6e, f). This finding reveals TMEM189 is a robust factor required for inhibition of FAR1 to render the cells resistant to ferroptosis.

To further validate the role of TMEM189 in ferroptosis, two independent TMEM189 sgRNAs were performed to suppress the expression of TMEM189. As shown in Fig. 6g, TMEM189 was barely undetectable in HT1080 cells expressing TMEM189 sgRNAs. As expected, loss of TMEM189 sensitized the cells to RSL3-induced ferroptosis (Fig. 6h). Moreover, this ferroptotic cell death was fully restored by ectopic expression of TMEM189 (Fig. 6i). Loss of TMEM189 in human glioblastoma U87MG also sensitized cells to ferroptosis (Supplementary Fig. S6a, b). In addition, endogenous FAR1 level is increased in TMEM189 KO cells (Supplementary Fig. S6c) and knocking out FAR1 in TMEM189 KO HT1080 cells rescued the ferroptosis sensitization (Supplementary Fig. S6d). In addition, over-expressing TMEM189 in GPX4−/− HT1080 cells induced decrease of FAR1 levels and delayed ferroptotic cell death upon Fer-1 withdrawal (Fig. 6k, i). Taken together, these data demonstrate that TMEM189 inhibits ferroptosis through decreasing FAR1 protein levels.

A very recent study by Zou et al. revealed the role of polyunsaturated ether phospholipids (PUFA-ePLs) in ferroptosis [34]. As PUFA has a very strong pro-ferroptotic effect, it is not surprising that PUFA-ePLs sensitize cells to ferroptosis on the whole no matter alkyl or vinyl bond in sn-1. To eliminate this possibility that PUFA in sn-2 might have a strong interference for the role of vinyl ether in sn-1, we treated the cells with C16(-O-)-18:1 PE and C18(plasm)-18:1 PE to test whether the vinyl-ether lipid inhibits SFA-induced ferroptosis. Consistent with our hypothesis, supplementation of C18(plasm)-18:1 PE not C16(-O-)-18:1 PE decreased FAR1 levels and efficiently suppressed SFA-mediated ferroptosis (Supplementary Fig. S6e, f). Additionally, previous reports showed that plasmalogens effectively protect PUFA-PLs from lipid peroxidation through the double bond [35–37]. Taken together, plasmalogens indeed play an important protective role in ferroptosis at least partially through inhibiting FAR1 expression.

To elucidate the functional consequence of TMEM189, we analyzed the data from GEPIA 2 (http://gepia2.cancer-pku.cn/) and found that TMEM189 expression is upregulated in almost all human cancers (Supplementary Fig. S7a). In accordance with this, TCGA database from cBioPortal for cancer genomics (https://www.cbioportal.org/) reveals TMEM189 exhibited high frequency of amplification in various human cancers (Supplementary Fig. 7,b). Moreover, Kaplan–Meier survival analysis from the TCGA database indicates high expression of TMEM189 is closely associated with poor prognosis in patients with kinds of cancers (Supplementary Fig. S7c). In addition, high level of TMEM189 is correlated with low expression of FAR1 and AGPS and high expression of GPX4 (Supplementary Fig. S7d). Together, these data imply that TMEM189 might facilitate tumorigenesis through ferroptosis resistance.

## Discussion

Lipids are important regulators of ferroptosis. The initiation and execution of cell death are intimately linked to lipid metabolism [14, 20–24, 38]. There are three major types of fatty acids: SFA, monounsaturated fatty acid (MUFA) and PUFA. Recent studies reveal that free PUFA or PUFA-phospholipids (PUFA-PLs) are oxidized through non-enzymatic oxidation by free radicals or enzymatic catalyzation of lipoxygenase during ferroptosis initiation [20–24]. The accumulation of peroxidized PUFA-PLs is sufficient for the execution of cell death, which can be prevented by ACSL4 [20]. Ferroptosis induced by GPX4 inhibition is dependent on the activity of ACSL4 by incorporating free PUFA into glycerophospholipids. Conversely, MUFA potently suppresses ferroptosis by opposing PUFA activation, which is dependent on ACSL3, another member of ACSL family (ACSL1, ACSL3-6). ACSL3 protects cells from ferroptosis by displacing PUFA with MUFA from PLs [38]. However, whether and how SFA impacts cell death sensitivity is less understood.

Our study highlighted a previously uncovered SFA-mediated ferroptotic pathway, distinct from the current ferroptosis model mainly focusing on PUFA oxidation. Here we reveal show that SFA drives cells into a state that makes them sensitive to ferroptosis induced by cystine starvation, system X_c_^-^ inhibition or GPX4 inactivation. We also revealed SFA-mediated ferroptotic cell death is dependent on FAR1 by converting SFA to fatty alcohol. Inactivation of FAR1 prompted the cells resistant to ferroptosis. Although numerous studies showed that mitochondria might be involved in ferroptosis [15, 25], the cells with the depletion of mitochondria were as sensitive to erastin and RSL3 as wild-type cells [39]. This implies mitochondria is not necessary for ferroptosis and other subcellular organelles might be essential for the initiation and execution of ferroptosis. Our findings indicate that FAR1-induced ferroptosis relies on the pathway of ether phospholipid production in peroxisomes and inhibition of peroxisomes de novo biogenesis largely abolished erastin and RSL3-induced ferroptosis, uncovering an important role of peroxisomes in ferroptosis.

Although the precise mechanisms by which ether phospholipids induce ferroptosis will still require further elucidation, our study emphasizes the importance of the pathway of ether lipid biosynthesis in the process of ferroptosis. One possible explanation of the role of ether phospholipids is SFA converted to fatty alcohol and then incorporated to sn-1 position of PLs containing PUFA in sn-2, enriched peroxidized ether-linked PUFA-PLs sensitized cells to ferroptosis. Moreover, we revealed a distinct role of vinyl ether lipid-plasmalogens generation in ferroptosis. A very recent publication by Zou et al. revealed PUFA-ePLs contribute to ferroptosis [34]. Most of their findings are highly consistent with ours, only one notable difference that they showed TMEM189 has no effect on ferroptosis while we found a protective role of TMEM189. To test whether the discrepancy results from the different cell lines used in these two studies, we knocked down TMEM189 in a panel of cancer cell lines using TMEM189 sgRNA. As shown in Supplementary Fig. S6a, HT1080 and U87MG cells exhibit robustly higher basal TMEM189 protein levels than 786-O, OVCAR-8 and HuH7 cells. In addition, we did not observe a significant difference in FAR1 protein levels among these cell lines except HUH7 cells in which FAR1 is undetectable. However, FAR1 protein levels are markedly increased in HT1080 and U87MG cell lines expressing TMEM189 sgRNA, while there is no increase in 786-O, OVCAR-8 and HuH7 cells. Notably, TMEM189 KO in HT1080 and U87MG cells sensitized cells to ferroptosis, while TMEM189 has no effect on protecting cells from ferroptosis in 786-O, OVCAR-8, and HuH7 cells (Supplementary Fig. S6b).

As there is low expression of TMEM189 in 786-O, OVCAR-8 and HuH7 cells, indicating that these cell lines have low ability to produce plasmalogens to inhibit FAR1 expression, so TMEM189 KO in these cell lines has little effect on FAR1 levels and ferroptosis sensitivity. These data indicate that cells with high levels of TMEM189 are more susceptible to ferroptosis than cells with low levels of TMEM189 when TMEM189 is silenced in these cell lines.

The function consequence of TMEM189 required for plasmalogen generation in multiple pathological conditions or diseases is little known. Through expression profile analysis of TMEM189 from the cancer databases, we found that TMEM189 is aberrantly accumulated in almost all types of human cancers and more importantly, the cancer patients expressing higher levels of TMEM189 have a significantly shorter overall survival compared to the patients bearing tumors expressing lower levels of TMEM189 expression (Supplementary Fig. S5c). Thus, these data suggest that TMEM189 overexpression is a critical means of tumorigenesis in human cancers. Although numerous studies indicate loss of GPX4 induced ferroptotic cell death in cancer cells [11, 24], deficiency of GPX4 also affects normal development or cell viability in normal tissue [16, 17]. In contrast to lethality induced by loss of GPX4, TMEM189-deficient mice survive normally as well as wild type [32]. Therefore, given the enrichment of TMEM189 in human cancers and poor clinical benefit with high TMEM189 expression, we propose TMEM189 as a very promising therapeutic target than GPX4 for activating ferroptosis in human cancers.

## Materials and methods

### Cell culture and stable cell lines

HT1080, 786-O, MDA-MB-231, U87MG, A2780, HO8910, HepG2, Hep3B, HuH6, HuH7, and Li-7 cancer cells were obtained from the Cell Bank of the Chinese Academy of Science (Shanghai, China) and have been proven to be negative for mycoplasma contamination. No cell lines used in this work were listed in the ICLAC database. All cells were cultured in a 37 °C incubator with 5% CO_2_ and maintained in culture medium supplemented with 10% fetal bovine serum (Biological Industries, Israel), 1% Penicillin/Streptomycin solution. HT1080, HO8910, U87, A2780, HepG2, HuH6 and HuH7 were cultured in Dulbecoo’s modified Eagle’s medium. 786-O, MDA-MB-231 and Li-7 were cultured in RPMI-1640 medium. Hep3B was cultured in Minimum Essential medium. HT1080 GPX4−/− cell line was a gift from Dr. Minghui Gao (Harbin Institute of Technology).

### Plasmids

For FAR1 and TMEM189, full-length FAR1 and TMEM189 were cloned into pcDNA3.1 (Invitrogen) and Plenti4 vectors. For GPX4, full length GPX4 was cloned into Pcin4-Flag-HA vector. For the sgRNAs of FAR1, TMEM189, PEX3, and PEX16, these sgRNAs were constructed to PX458 and plenti-CRISPR-V2 vectors.

### Chemicals

Erastin (S7242), RSL3 (S8155), ML210 (S7088), Ferrostatin-1 (S7243) were pursued from Selleck. acid (P0500), 1-hexadecanol (258741), stearic acid (S4751), 1-Octadecanol (258768), and TBH (458139) were pursued from Sigma Aldrich.

### CRISPR-Cas9 and shRNA-mediated gene silence

The sgRNA sequences were designed from the website (http://crispor.tefor.net/). sgFAR1: CGAACTCACCCAACCTAAAC, AGTAGTCTATCCACCACCTG; sgTMEM189: GCGATGGTGTTTACGTGGC, GTGCGACCACTTGTGGATC; sgPEX16: ATGTACAAAAACTCTGCGA, TGCCGTCCCTCCCGCTGCT; sgPEX19: AGCTCTTCTTCCGACATGC, GGGCTAGGCATGGACGAAG. For shRNA-mediated gene knockdown, the following sequences were used: shFAR1: CCGGGCAAGAAATATCTGGTACTTTCTCGAGAAAGTACCAGATATTTCTTGCTTTTTTG, CCGGGCTGTTCAGTTAAATGTGATTCTCGAGAATCACATTTAACTGAACAGCTTTTTTG; shGNPAT: CCGGGCCAAGACATTGACTCCTAAACTCGAGTTTAGGAGTCAATGTCTTGGCTTTTTG, CCGGCCAGAAAGATTCTCTCTGAAACTCGAGTTTCAGAGAGAATCTTTCTGGTTTTTG; shAGPS: CCGGGCATCCTTAAATCCTAGTGATCTCGAGATCACTAGGATTTAAGGATGCTTTTTG, CCGGCCTCAGGTTTCCTCTATCTTTCTCGAGAAAGATAGAGGAAACCTGAGGTTTTTG.

### Endogenous metabolites screen

786-O cells were plated in 96-well plates at 5000 cells per well and pre-incubated with indicated endogenous metabolites library from Selleck. Then 16 h after pre-incubation of the metabolites, the cells were treated with 100 nM RSL3 for 24 h. The cell viability was measured using CCK8 and data were collected. For metabolites screen analysis, the metabolites which alone induced cell death were excluded. The data: (OD value of RSL3 treated cells-OD value of blank well)/(OD values of DMSO treated cells- OD value of blank well) were calculated and compared to the group without pre-treatment of the metabolites. The value with average log2(fold change) > 1 or < −1 (indicating fold > 2 or fold < −2) and average −log10(*P* value) > 2 (indicating *P* < 0.01) are used for analyzing.

### Cell viability assay

For cell viability assay, the cells were seeded at 5000 cells per well in 96-well plates. 16 h after seeding, the cells were treated with indicated concentration of RSL3. 24 h later, the plates were incubated with CCK8 for 1 h and read at 450 nm. The collected values were normalized to blank well. Then the relative cell viability was normalized to the respective DMSO-treated wells. Graphpad Prism 8 software was used to plot the regression fit curves.

### Cell death assay

For cell death analysis, cells were treated, collected, and initially stained with specific antibodies, then resuspended in PBS containing 1 μg/ml propidium iodide (PI) (P1304, Thermo Fisher Scientific) for 15 min, and washed twice. Then the cells were run on a flow cytometer and data were collected to analyze.

### Lipid peroxidation assay

Cells were harvested and washed with PBS, then resuspended with PBS containing 5 μM C-11 BODIPY dye (D3861, Thermo Fisher Scientific) and incubated in the tissue culture incubator for 30 min. Cells were then washed twice with PBS followed by resuspending in 200 μl PBS. ROS levels were analyzed using a Becton Dickinson FACS Calibur machine through the FL1 channel, and the data were analyzed using FlowJo. In each sample, 5000 cells were analyzed.

### Western blotting and antibodies

Cellular proteins were extracted using BC-100 lysis buffer supplemented with 1% protease inhibitor cocktail, and protein concentration was determined by a Bradford protein assay kit (Beyotime, China). Total 20 µg proteins were separated by 10% SDS-polyacrylamide gel, transferred onto nitrocellulose filter membrane (Millipore Corp, Billerica, MA, USA), blocked with 5% fat-free milk for 1 h at room temperature. The primary antibodies against FAR1(1:3000; A16284; ABclone), GPX4(1:1000; ab125066; abcam), ACSL4(1:1000; ab155282; abcam), TMEM189(1:1000; GTX32926; GeneTex), 4-HNE(1:1000; ab46545; abcam), Pex3 (1:1000; 10946-1-AP; Proteintech), Pex16 (1:1000; 14816-1-AP; Proteintech), Pex19 (1:1000; 14713-1-AP; Proteintech), FLAG M2(1:1000; F1804; Sigma-Aldrich), Vinculin(1:1000; 66305-1; Proteintech) and GAPDH(sc-47724; Santa Cruz Biotechnology) were incubated overnight at 4 °C, sequentially the peroxidase affinipure goat anti-rabbit or mouse IgG(H + L) (Jackson ImmunoResearch Laboratories) and enhanced chemiluminescence solution(GE Healthcare) were used for visualizing protein expression.

### Kidney IRI

8-week old female mice were purchased from Charles River Laboratories and prepared for the establishment of kidney ischemia/reperfusion. The mice were randomly distributed and anaesthetized with subcutaneous injection of sodium pentobarbital (30 mg/kg, Sigma). The heating pads were used to maintain the body temperature. Via the abdominal approach, the bilateral renal pedicles were clamped for 30 min by using a vascular clamp. Then the clamp was removed and the abdominal cavity was closed using sutures. For the group of ferrostatin-1 treatment, mice were injected intraperitoneally 200ul PBS containing 5 mg/kg ferrostatin-1 30 min before ischemia. All the mice were euthanized 24 h after IRI operation, and fresh kidneys were collected and embedded in 4% paraformaldehyde for further study. 6 mice in each group were used. The study is compliant with all relevant ethical regulations for animal experiments. All the experimental protocols were approved by the Institutional Animal Care and Use Committee of Shandong University.

### Patient specimens and immunohistochemistry, immunofluorescence

Hepatocellular carcinoma tissue microarray was purchased from Wuhan Google Biotechnology Co., Ltd, and human ductal breast cancer specimens were obtained from Qilu Hospital, Shandong University. The study complied with the ethical requirements of Shandong University. The kidney tissues embedded in paraffin were sliced into 4 µm thickness section for HE staining, Immunohistochemistry, immunofluorescence.

Immunohistochemistry was carried out to explore the relevance of FAR1 and 4-HNE in cancers and kidney IRI. Briefly, sections were soaked in xylene for dewaxing and graded alcohol for rehydrating, followed by incubated with 2% Tween-20 for 20 min. Antigen retrieval was performed by placing in citrate buffer in water bath for 15 min at 98 °C to shelter endogenous peroxidase activity, and 5% BSA was added to block nonspecific epitopes. Sections were incubated with primary antibodies against FAR1(1:500) and 4-HNE (1:200) in a humidified chamber at 4 °C overnight. Sections were cultured with biotin-labeled secondary antibody and then SABC reagent for 30 min according to the manufacturer’s instructions. 3’3-diaminobenzidine and DAPI dye were used for antigen detection. The immunohistochemistry images were photographed by Multispectral fully electric scanning microscopy imaging system (TissueGnostics, Austria), and calculated mean optical density (MOD) values by Image Pro-plus software.

Immunofluorescence was performed to focus on the relationship of FAR1 and TfR1 in kidney IRI, similar to immunohistochemistry procedure. Sections were incubated individually with FAR1(1:500) and TfR1(1:250; AB-2533029; Invitrogen), and then visualized by goat polyclonal Secondary Antibody to Rabbit IgG—H&L (Alexa Fluor^®^ 488) (1:200; ab150077; abcam) and DAPI dye.

### Preparation of phospholipids

Phospholipids as 5 mg/mL solutions in chloroform were purchased from Avanti Lipids (C16(-O-)-20:4 PC 878113, C16(-O-)-18:1 PE 878130, C18(plasm)-18:1 PE). Chloroform was evaporated under vacuum and lipids were reconstituted with ethanol. The stocks were then prepared with 20% 2-hydroxypropyl-beta-cyclodextrin (Cayman 16169) in PBS solution. Stocks were incubated at 37 °C for 30 min and then sonicated to clear solutions at room temperature.

### Statistics and reproducibility

Western blot, imaging results were independently repeated at least twice. For H&E and IHC assays, at least four sample size was used. All the other experiments were independently repeated at least three times. Statistical analysis was carried out using Microsoft Excel software and GraphPad Prism to assess the differences between experimental groups. Statistical significance was determined by using a two-tailed, unpaired Student’s *t*-test with a confidence interval (CI) of 95%. The variance is similar between the compared groups. *P* ≤ 0.05 was denoted as statistically significant. Statistical analysis of all survival curves data was performed using log-Rank (Mantel-Cox) test. No data were excluded from the study. For in vitro cell-based experiments, the investigators were not blinded during data acquisition and analysis. The application of treatments and processing procedures made it difficult for blinding but there was no human bias given all the data were collected independently using instrumentation. For the animal experiments, the investigators were not blinded to the group allocation. However, at least two observers measured lipid peroxidation to alleviate human bias in these data.

## Supplementary information

Supplementary Figure legends

Supplementary Figure 1

Supplementary Figure 2

Supplementary Figure 3

Supplementary Figure 4

Supplementary Figure 5

Supplementary Figure 6

Supplementary Figure 7

Dataset 1

## Data Availability

The survival curve and gene correlation data of FAR1 and TMEM189 gene expression data in human cancer tissues were derived from TCGA database and in human cancer cell lines from CCLE (https://portals.broadinstitute.org/ccle/). The TMEM189 expression levels correlated with ferroptosis sensitivity were derived from CTRP V2 (http://portals.broadinstitute.org/ctrp/). The TMEM189 gene expression data in human cancer tissues were derived from GEPIA (http://gepia2.cancer-pku.cn/). The alteration frequency of TMEM189 gene were derived from CbioPortal (https://www.cbioportal.org/). All the data supporting the findings of this study are available from the corresponding author on reasonable request.
