# Supplementary material for: Peroxisome-driven ether-linked phospholipids biosynthesis is essential for ferroptosis
Source: Cell Death Differ. 2021 Mar 17;28(8):2536–51. doi: 10.1038/s41418-021-00769-0 (PMC8329287; doi:10.1038/s41418-021-00769-0)
Supplement: Supplementary file 1 — Supplementary Figure legends [file 41418_2021_769_MOESM1_ESM.docx]

**Supplementary Figure Legends**

**Figure S1. Long-chain saturated fatty acids and fatty alcohol promotes ferroptosis susceptibility.**

**a**, Dose-dependent toxicity of 1-hexadecanol induced cell death of 786-O and HT1080 cells treated with RSL3 (200nM). Cell viability was assessed 24h thereafter using CCK8.

**b**, Cell death measurement of 786-O cells pre-incubated with 1-hexadecanol (20uM) for 12h were treated with RSL3 (200nM) and Fer-1 (1uM) for 12h.

**c**, cell death measurements of HT1080 and 786-O cells pre-incubated with PA (20uM) or 1-HE (20uM) treated with erastin (2uM) and Fer-1 (1uM) for 12h. PA, palmitic acid. 1-HE, 1-hexadecanol.

**d**, cell death measurements of HT1080 and 786-O cells pre-incubated with PA (20uM) or 1-HE (20uM) treated with RSL3 (200nM) and Fer-1 (1uM) for 12h.

**e**, cell death measurements of HT1080 cells and 786-O pre-incubated with SA (20uM) or 1-Oct (20uM) treated with erastin (2uM) and Fer-1 (1uM) for 12h. SA, stearic acid. 1-OE, 1-Octadecanol.

**f**, cell death measurements of HT1080 and 786-O cells pre-incubated with SA (20uM) or 1-Oct (20uM) treated with RSL3 (200nM) and Fer-1 (1uM) for 12h. Data and Error bars are mean ± s.d., n=3 **(a-f**) independent repeats. All P values were calculated using two-tailed unpaired Student’s t-test.

**Figure S2. The pathway of ether phospholipid biosynthesis sensitizes the cells to ferroptosis.**

**a**, A gene list of peroxisomes involved in ferroptosis from KEGG analysis in Fig. 2**a**.

**b**, A gene list of peroxisomes and ether lipid synthesis process involved in ferroptosis from GO analysis in Fig. 2**b**.

**c**, Schematic diagram of peroxisome-driven initial ether phospholipid synthesis.

**d**, Volcano plots showing peroxisome-related genes enrichment between DMSO and ML210 treated 786-O cells which are transfected with a genome-wide CRISPR-CAS9 sgRNA lentivirus pools.

**e**, A model of peroxisomes *de novo* biogenesis.

**f**, Schematic diagram of the alkyl and vinyl-ether lipids.

**g,** Dose-dependent toxicity of RSL-3 induced cell death of 786-O and HT1080 cells expressing sh-ctrl or sh-GNPAT. Cell viability was assessed 24h thereafter using CCK8.

**h,** qRT-PCR analysis of GNPAT mRNA levels in HT1080 and 786-O cells expressing sh-ctrl or sh-GNPAT.

**i**, Cell viability of 786-O and HT1080 cells expressing sh-ctrl or sh-AGPS after 24h RSL3 treatment.

**g**, qRT-PCR analysis of AGPS mRNA levels in HT1080 and 786-O cells expressing sh-ctrl or sh-AGPS. Data and Error bars are mean ± s.d., n=3 **(g-j**) independent repeats. All P values were calculated using two-tailed unpaired Student’s t-test.

**Figure S3. Alkyl ether lipids render cancer cells susceptible to ferroptosis.**

**a**, Fluorescent imaging analysis of PTS1-GFP distribution in HT1080 cells expressing sg-ctrl, sg-PEX16 or sg-PEX19.

**b**, WT and FAR1 KO HT1080 cells were pre-incubated with C16(-O-)-20:4 PC (1uM) for 24h, and then treated with RSL3(100nM) for 8h.

**Figure S4. FAR1 functions as a potential tumour suppressor.**

**a**, FAR1 mRNA expression levels in the indicated types of cancer cell lines from Cancer Cell Line Encyclopedia (CCLE) database.

**b**, Dot plot depicting the correlation of the dependency of a cell on FAR1 and the expression level of AGPS or ACSL4 in a panel of 559 different cancer cell line (DepMap; <https://depmap.org/portal/> ). Cell lines showed high co-dependence of FAR1, AGPS and ACSL4.

**c**, Dot plot showing the correlation of the expression level of FAR1 and AGPS, GNPAT, ACSL4 and GPX4 in the colorectal tumor samples from the TCGA dataset.

**d**, Dot plot showing the correlation of the expression level of FAR1 and AGPS and GPX4 in the breast tumor samples from the TCGA dataset.

**e**, Kaplan–Meier survival curves for patients with colorectal or breast cancers containing

low or high expression of FAR1 mRNA in the TCGA dataset. The P value was calculated using the log-rank Mantel–Cox test.

**f**, FAR1 mRNA expression levels in the indicated stage of Tumor Nude Metastasis (TNM) from TCGA breast cancer dataset. P values were calculated using two-tailed unpaired Student’s t-test.

**g**, Kaplan–Meier survival curves for patients with indicated tumour types containing

low or high expression of FAR1 mRNA in the TCGA dataset. The P value was calculated using the log-rank Mantel–Cox test.

**Figure S5. FAR1 protein level is elevated during renal IR.**

**a**, Immunofluorescence staining of FAR1 and TFR1 in the cortical renal tissues from the mice after ischaemia reperfusion (IR) or sham treatment. The experiment was repeated three times, independently, with similar results.

**b-c**, qRT-PCR analysis of TMEM189 mRNA levels in U2OS, HT1080 and 786-O cells treated with erastin and RSL3 for the indicated time.

**d,** A model of alkyl and vinyl-ether phospholipids synthesis pathway. Data and Error bars are mean ± s.d., n=3 **(b-c**) independent repeats. All P values were calculated using two-tailed unpaired Student’s t-test.

**Figure S6. TMEM189 protects cells from ferroptosis by inhibiting expression of FAR1.**

**a**, Western blot analysis of cancer cell lines expressing sg-ctrl or sg-TMEM189. The Western blot experiments were repeated twice, independently, with similar results

**b**, Cell death measurement of cancer cell lines expressing sg-ctrl or sg-TMEM189 treated with RSL3(100nM) for 8h.

**c**, Western blot analysis of HT1080 cells expressing indicated sgRNA.

**d**, Cell death measurement of cells from (**c**) expressing sg-ctrl or sg-TMEM189 treated with RSL3(100nM) for 8h.

**e,** Western blot analysis of HT1080 cells supplemented with C16(-O-)-18:1 PE and C18(plasm)-18:1 PE for 48h.

**f**, HT1080 cells were pre-incubated with C16(-O-)-18:1 PE and C18(plasm)-18:1 PE for 48h, were treated with RSL3 (100nM) for 12h.

**Figure S7. TMEM189 is a novel potential target for cancer therapy.**

**a**, TMEM189 mRNA expression levels in the indicated types of normal and tumour tissues from GEPIA 2 database.

**b**, Genomic alteration frequency of TMEM189 in a series of cancer types showing TMEME189 trends to amplification from the TCGA database. The data was collected from the cBioPortal (<https://www.cbioportal.org/>).

**c**, Kaplan–Meier survival curves for patients with indicated types of cancers containing

low or high expression of TMEM189 mRNA in the TCGA dataset. The P value was calculated using the log-rank Mantel–Cox test.

**d**, Dot plot showing the correlation of the expression level of TMEM189 and FAR1, AGPS and GPX4 in the breast tumor samples from the TCGA dataset.
