# Supplementary figures and images for: Peroxisome-driven ether-linked phospholipids biosynthesis is essential for ferroptosis
Source: Cell Death Differ. 2021 Mar 17;28(8):2536–51. doi: 10.1038/s41418-021-00769-0 (PMC8329287; doi:10.1038/s41418-021-00769-0)

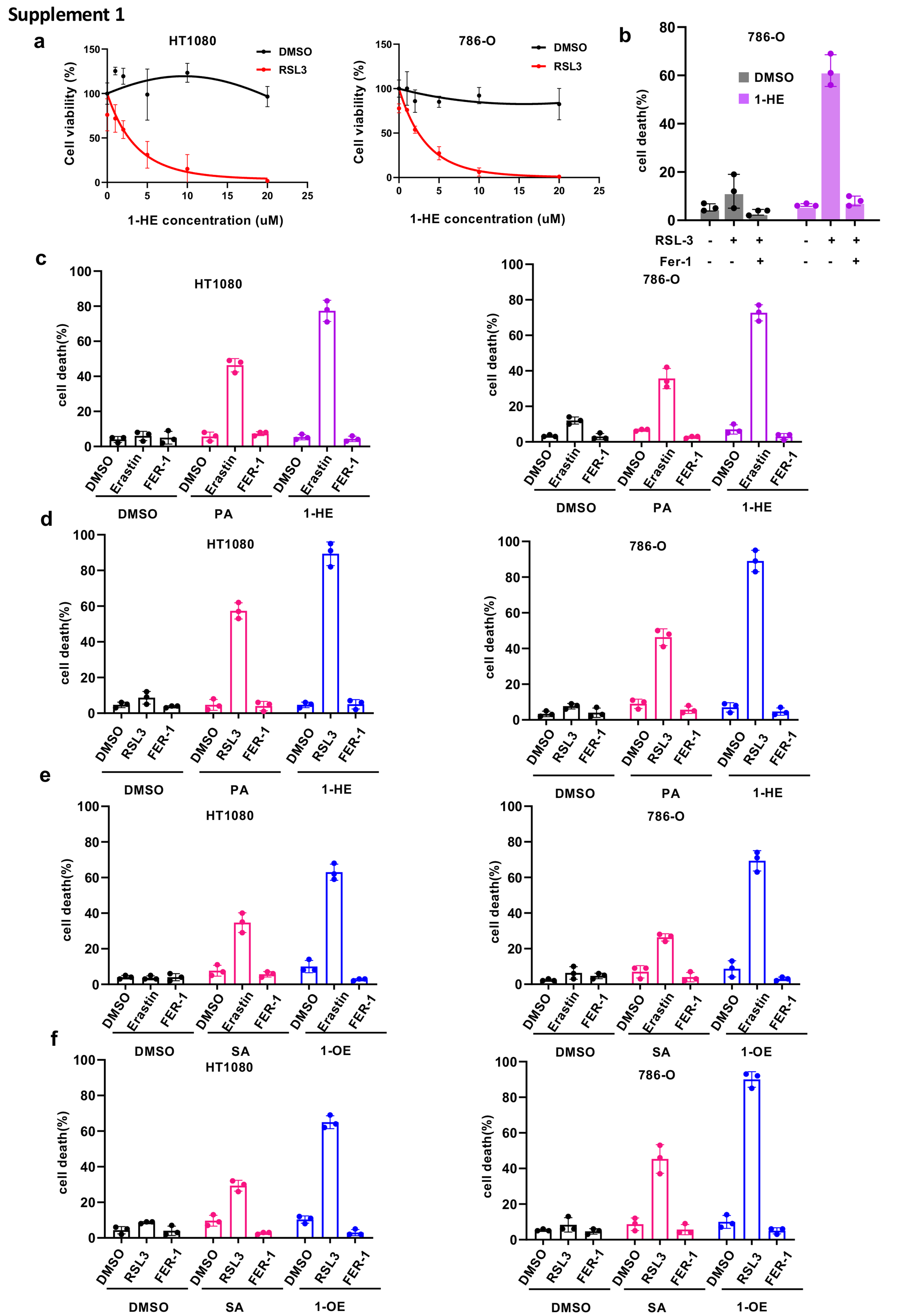

Supplement: Supplementary file 2 — Supplementary Figure 1 [file 41418_2021_769_MOESM2_ESM.tif]

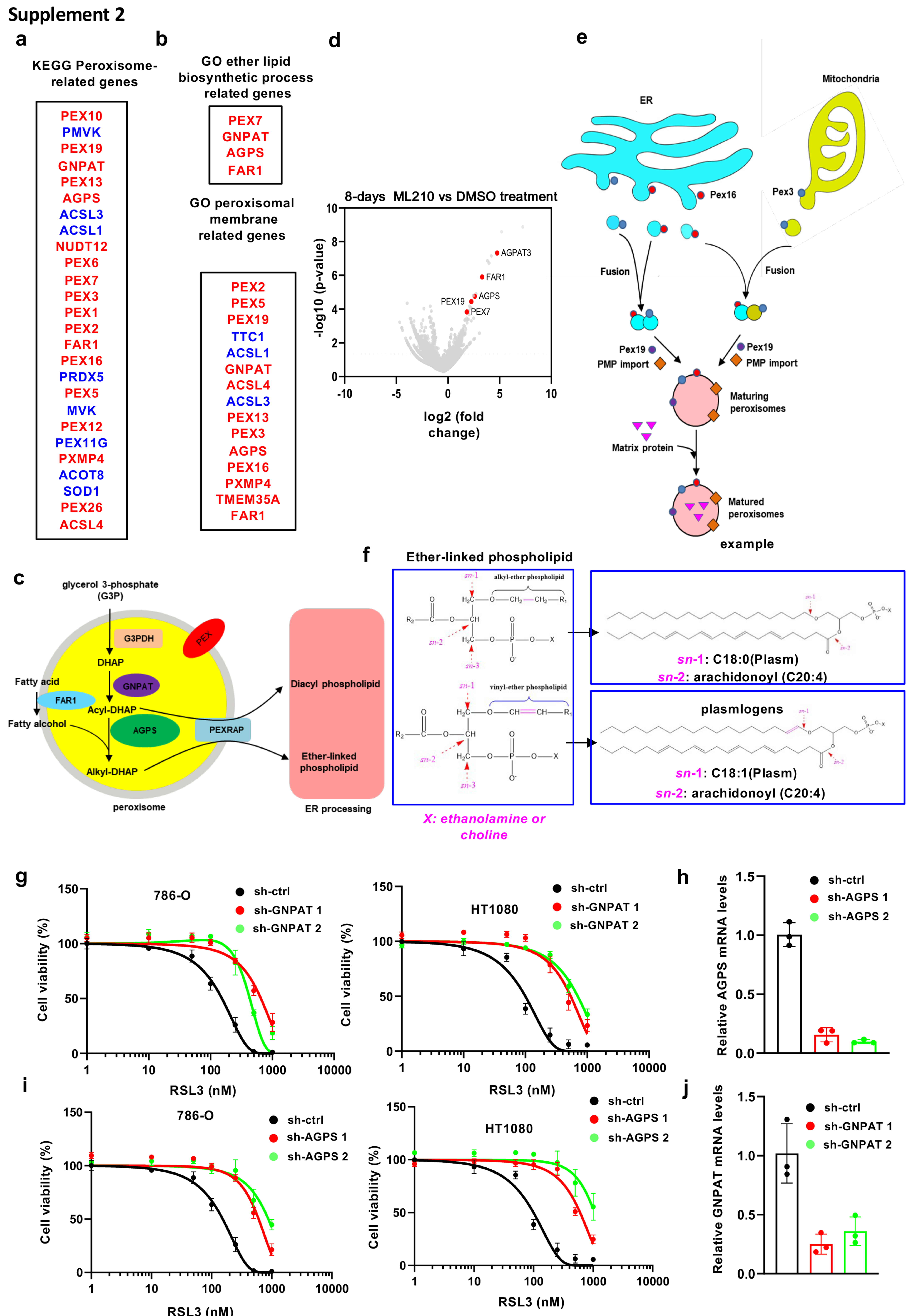

Supplement: Supplementary file 3 — Supplementary Figure 2 [file 41418_2021_769_MOESM3_ESM.tif]

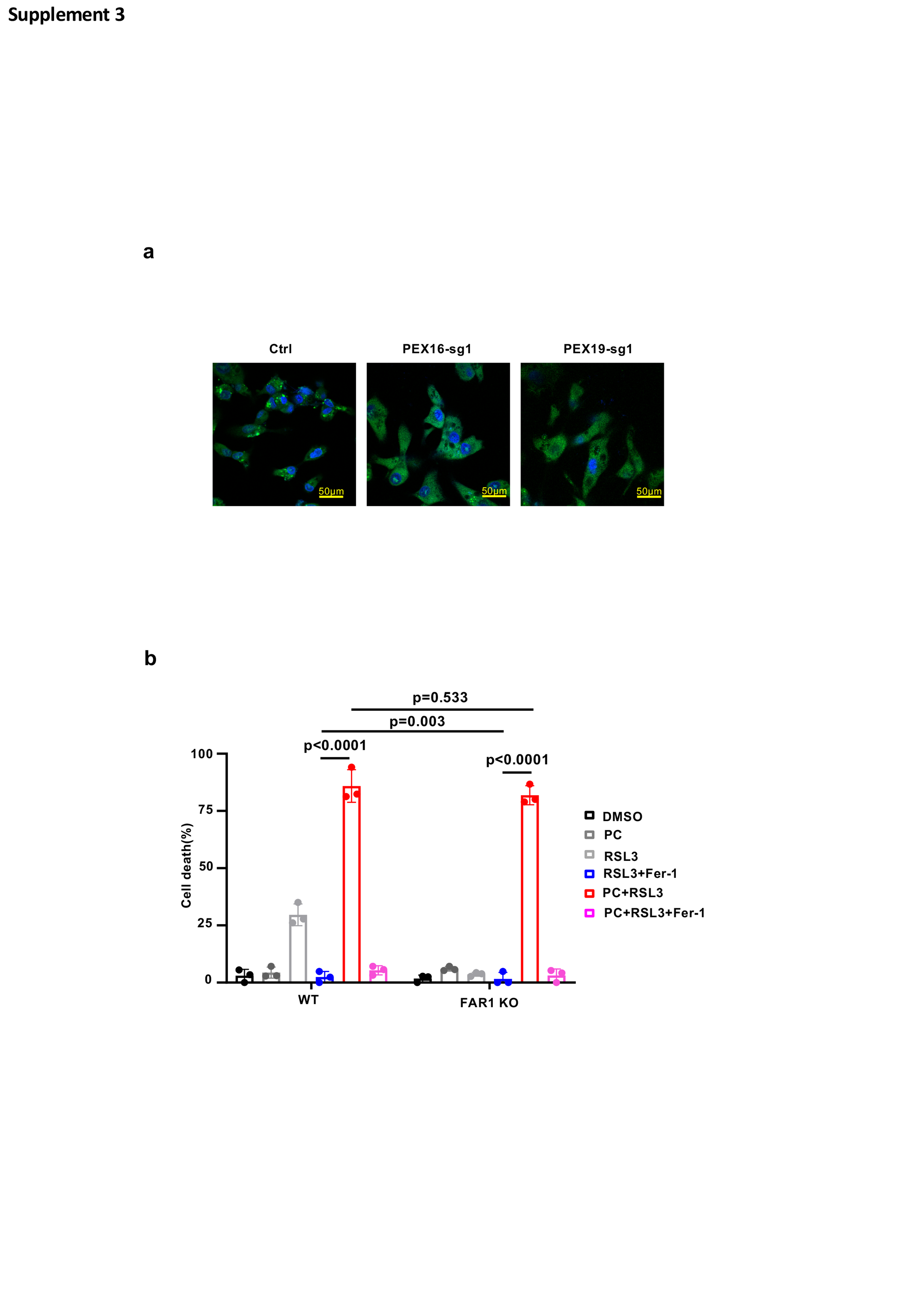

Supplement: Supplementary file 4 — Supplementary Figure 3 [file 41418_2021_769_MOESM4_ESM.tif]

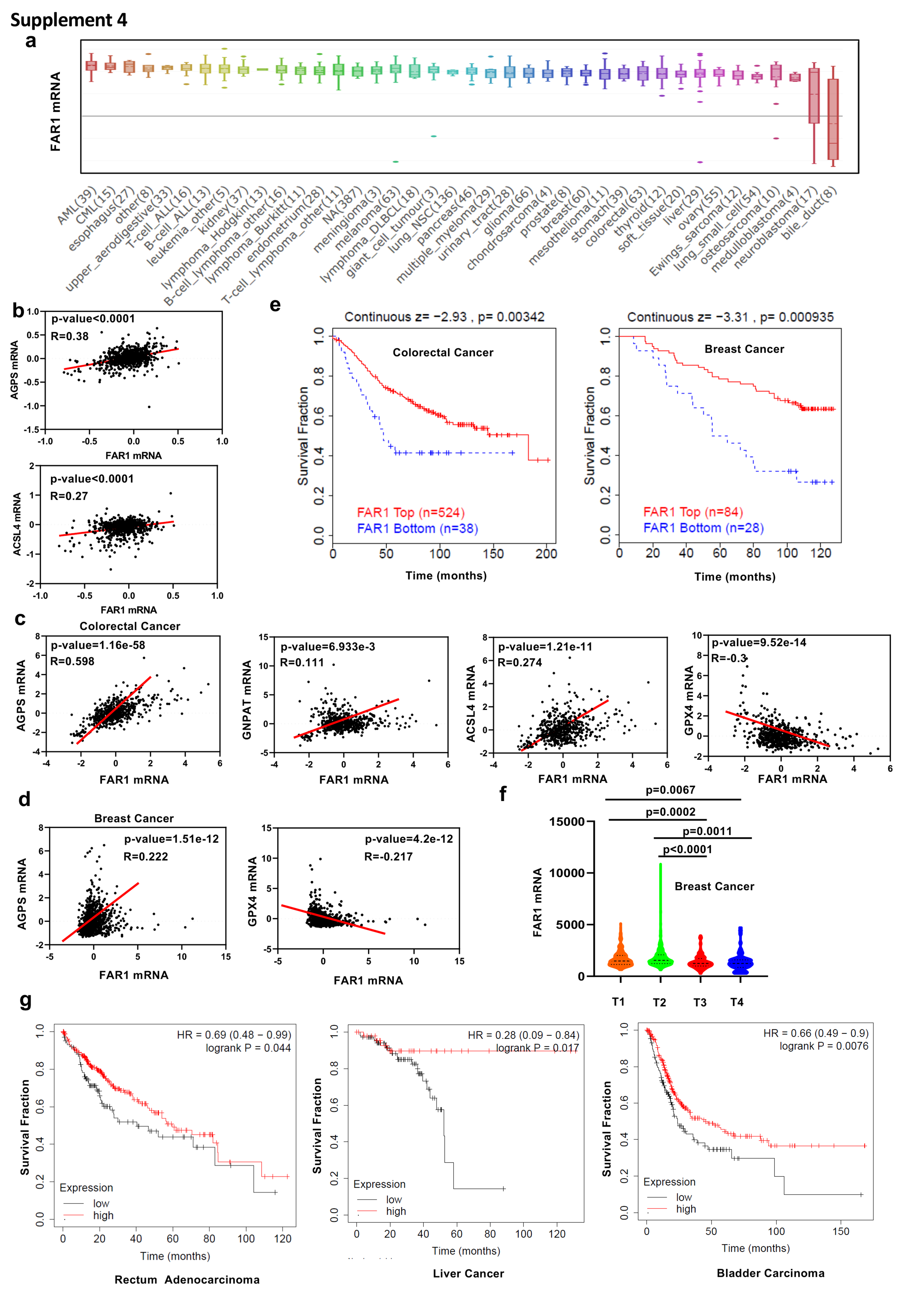

Supplement: Supplementary file 5 — Supplementary Figure 4 [file 41418_2021_769_MOESM5_ESM.tif]

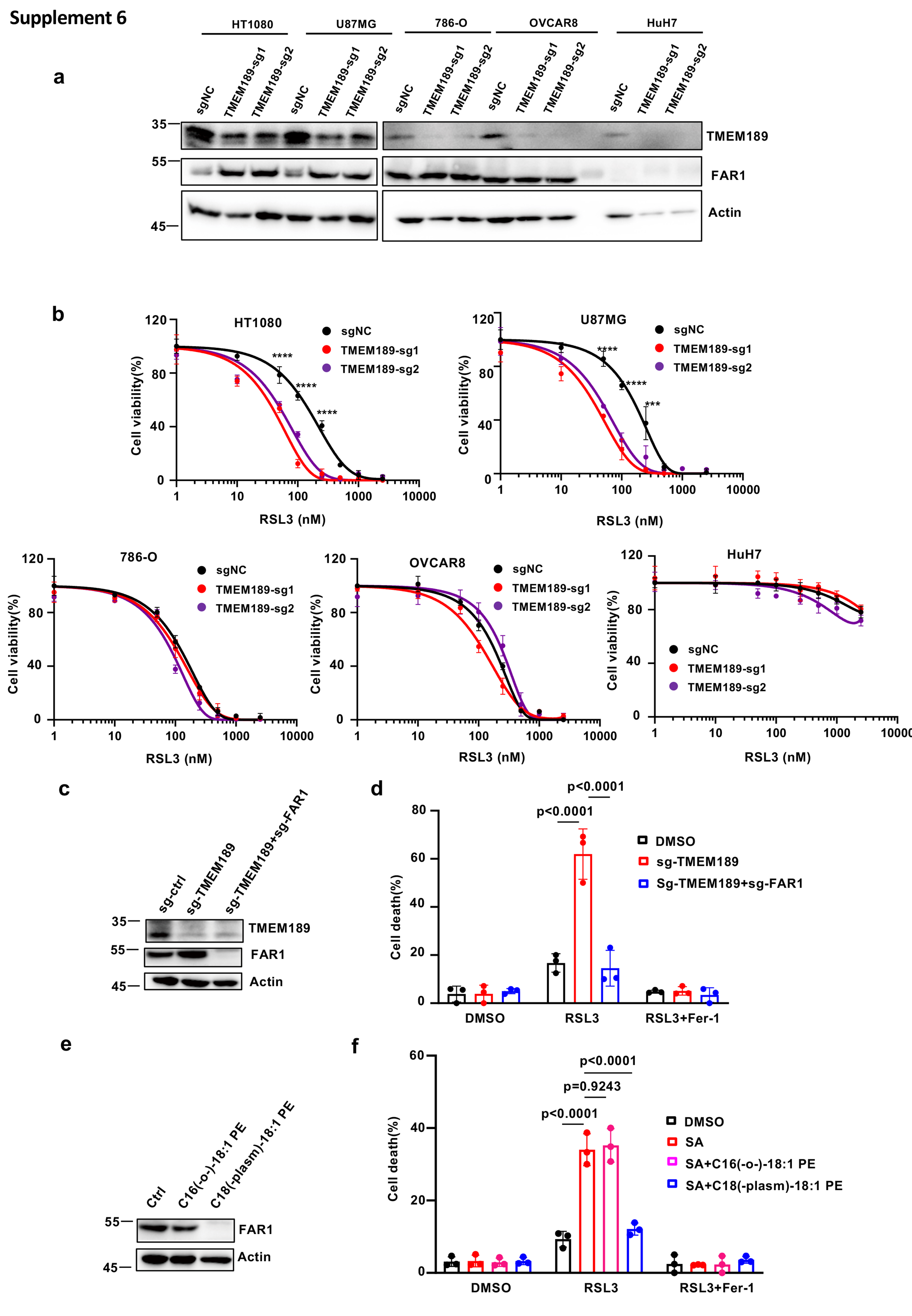

Supplement: Supplementary file 7 — Supplementary Figure 6 [file 41418_2021_769_MOESM7_ESM.tif]
